# Factors affecting COVID-19 infected and death rates inform lockdown-related policymaking

**DOI:** 10.1371/journal.pone.0241165

**Published:** 2020-10-23

**Authors:** Satyaki Roy, Preetam Ghosh

**Affiliations:** 1 Department of Genetics, University of North Carolina, Chapel Hill, North Carolina, United States of America; 2 Department of Computer Science, Virginia Commonwealth University, Richmond, Virginia, United States of America; University of Louisville, UNITED STATES

## Abstract

**Background:**

After claiming nearly five hundred thousand lives globally, the COVID-19 pandemic is showing no signs of slowing down. While the UK, USA, Brazil and parts of Asia are bracing themselves for the second wave—or the extension of the first wave—it is imperative to identify the primary social, economic, environmental, demographic, ethnic, cultural and health factors contributing towards COVID-19 infection and mortality numbers to facilitate mitigation and control measures.

**Methods:**

We process several open-access datasets on US states to create an integrated dataset of potential factors leading to the pandemic spread. We then apply several supervised machine learning approaches to reach a consensus as well as rank the key factors. We carry out regression analysis to pinpoint the key pre-lockdown factors that affect post-lockdown infection and mortality, informing future lockdown-related policy making.

**Findings:**

Population density, testing numbers and airport traffic emerge as the most discriminatory factors, followed by higher age groups (above 40 and specifically 60+). Post-lockdown infected and death rates are highly influenced by their pre-lockdown counterparts, followed by population density and airport traffic. While healthcare index seems uncorrelated with mortality rate, principal component analysis on the key features show two groups: states (1) forming early epicenters and (2) experiencing strong second wave or peaking late in rate of infection and death. Finally, a small case study on New York City shows that days-to-peak for infection of neighboring boroughs correlate better with inter-zone mobility than the inter-zone distance.

**Interpretation:**

States forming the early hotspots are regions with high airport or road traffic resulting in human interaction. US states with high population density and testing tend to exhibit consistently high infected and death numbers. Mortality rate seems to be driven by individual physiology, preexisting condition, age etc., rather than gender, healthcare facility or ethnic predisposition. Finally, policymaking on the timing of lockdowns should primarily consider the pre-lockdown infected numbers along with population density and airport traffic.

## 1 Introduction

Epidemics and pandemics have marked human history since time immemorial. Over the course of the last millennium, outbreaks such as plague, flu and Ebola have globally claimed millions of lives [1]. COVID-19 is the latest pandemic that, since its inception in China in November 2019, has redefined every facet of human life. As of June 2020, 470,000 lives have been lost, and there is a looming possibility of a still higher fatality in Brazil, the UK, USA and parts of Asia [2].

Most countries seemed discernibly ill-equipped to handle an outbreak of a mammoth proportion like COVID-19. In the absence of credible vaccination treatment [3], social distancing and ensuing lockdown efforts, which were intended to curb infection spread, are threatening to bring the global economy to a halt. The decline in industrial output and stock exchange percentage, increase in the price of goods [4] as well as a projected contraction in US GDP [5] are prompting the national administrations to relax lockdown rules and revive global economy. Currently we are experiencing a resurgence in new cases and deaths due to COVID-19, which several epidemiologists term an extension of the first wave itself (and not the “second” wave). In the US, the epicenter has shifted from New York and New Jersey to Arkansas, Arizona and South Carolina [6], while on the world map, the infected and death numbers continue to rapidly soar in Beijing, India and Japan [7–9].

The absence of prior knowledge and coordinated mitigation strategies have not only worsened the threats posed by COVID-19, but also stymied research efforts on its clinical, epidemiological or socioeconomic implications [3, 10]. Dearth and inaccuracy in testing, reluctance in reporting death and recovery [11] and dubious information in print and social media [12] further misguide precautionary and control measures. Keeping these issues in mind, robust predictions on the social, cultural, demographic, health, environmental factors affecting infection and death are of interest to epidemiologists, environmentalists, pharmacists and government policymakers [13–15].

There have been several attempts to apply machine learning (ML) and artificial intelligence techniques to study the global phenomenon COVID-19 from the following two standpoints: (1) clinical and (2) epidemiological data analysis and prediction modeling. First, on the clinical front, efforts have been made to develop prediction models [16] and therapeutic approaches to identify the vulnerable individuals based on genetic and physiological predispositions [17, 18] or image-processing on clinical reports [19]. Second, the epidemiologists are attempting to exploit ML approaches to understand the spread dynamics of COVID-19. Inga Holmdahl et al. [20] explained the pitfalls and usefulness of data-driven forecasting models that make predictions through curve fitting or mechanistic models that simulate epidemic spreads. The existing forecasting models attempt to apply supervised and unsupervised ML to trace the trends in infection dynamics [21] or neural networks (such as recurrent neural networks) to project the new infections over time [22]. Golestaneh et al. performed logistic modeling on a cohort of 505,992 ambulatory care patients hospitalized during pre- and post-COVID periods to show that the odds of mortality of whites and blacks are statistically equivalent [23]. Myers et al. analyzed the COVID-19 positive patients in California to investigate its prognosis in the higher age groups and individuals with preexisting conditions [24]. Zoabi et al. applied ML on 51,831 COVID-19 positive patients to understand the effect of gender, age and contact to show that close social interaction is a strong feature for COVID-19 transmissibility [25]. Khan et al. applied regression tree, cluster analysis and principal component analysis on Worldometer infection count data to study the variability and effect of testing in prediction of confirmed cases [26]. Finally, Pan et al. studied the effects of the myriad public health interventions (such as lockdown, traffic restriction, social distancing, home quarantine, centralized quarantine, etc.) on 32,583 COVID-19 patients, with respect to their age, sex, residential location, occupation, and severity [27].

*Contributions*: While it is evident that factors such as gender, race, age, testing, social contact and distancing have been analyzed in a piecemeal manner, there is no comprehensive study that combines the demographic, economic, and epidemiological, ethnic and health indicators for infection and mortality from COVID-19. To address this gap, we carry out a machine learning-based analysis with the following three objectives.

1. We curate a dataset of diverse features (detailed in Sec. 2.1) from 50 states of USA. This dataset is somewhat unique, since, in addition to the above features, it includes factors such as airport traffic, homeless and variations in lockdown dates. Also, note that the lockdown was enforced on the US states at around the same time, when each state was at a different stage of the COVID-19 infection cycle.
2. We analyze the variation of COVID-19 infection spread and mortality rates using a set of standard supervised ML methods. We rank the key discriminatory factors based on the importance score calculated from randomized decision trees. We combine the findings to identify the most vulnerable age groups and US states. We also show the effect of testing and lockdowns on the infection spread dynamics.
3. We utilize multiple linear regression to gauge the extent to which the key pre-lockdown factors affect the post-lockdown infected and death numbers. This study assigns weights to features and drive mitigation efforts and large scale policymaking.

Our data-driven experiments using supervised methods demonstrate that population density, testing [28] and airport traffic [29] are key factors contributing to infection and mortality rates. Furthermore, high age group (40 and beyond, and specifically exceeding 60) population are more vulnerable. Principal component analysis on the key features show two groups: highly affected US states (1) forming early epicenters and (2) showing consistent or newly peaking rate of infection and death. Multiple regression analysis shows that the post-lockdown numbers are most influenced by the pre-lockdown infected and death numbers followed by population density and airport activity, while overall healthcare index of a state does not seem to play a part in the overall death count. Similarly, the race of individuals did not play any significant role in the infection or mortality numbers. Despite increased testing rates, the fraction of individuals tested positive drop approximately three weeks into the lockdown, suggesting that the social distance measures has had an impact on curbing spread. Finally, we discuss the role of mobility and distance in infection spread. In the absence of large-scale inter-state mobility data, our case study on the boroughs of New York City show that peaks of infection correlate better with inter-zone mobility than the inter-zone distance.

## 2 Materials and methods

All the experiments have been performed using Scikit-learn, which is a popular Machine Learning library in Python [30].

### 2.1 Dataset

Let us discuss the details of the two datasets used in this work.

#### 2.1.1 Data from US states

Our dataset has been carefully curated from several open sources to examine the possible factors that may affect the COVID-19 related infection and death numbers in the 50 states of USA. The individual open-access data sources as well as the integrated (curated) dataset has been shared on GitHub (https://github.com/satunr/COVID-19/tree/master/US-COVID-Dataset). Below, we discuss a summary of the features and output labels of the integrated dataset.

- *Gross Domestic Product* (in terms of million US dollars) for US states [31] (filename: source/GDP.xlsx, feature name: GDP).
- *Distance* from one state to another (is not measured in miles but the euclidean distance between their latitude-longitude coordinates between the pair of states [32]) (filename: source/Data_distance.xlsx, feature name: *d*(*state*1, *state*2)).
- *Gender* feature(s) is a fraction of total population representing the male and female individuals [33] (filename: source/Data_gender.csv, feature name: Male, Female).
- *Ethnicity* feature(s) are the fraction of total population representing white, black, Hispanic and Asian individuals (we leave out other smaller ethnic groups) [34] (filename: source/Data_ethnic.csv, feature name: White, Black, Hispanic and Asian).
- *Healthcare index* is measured by Agency for Healthcare Research and Quality (AHRQ) on the basis of (1) type of care (like preventive, chronic), (2) setting of care (like nursing homes, hospitals), and (3) clinical areas (like care for patients with cancer, diabetes) [35] (filename: source/Data_health.xlsx, feature name: Health).
- *Homeless* feature is the number of homeless individuals of a state [36] (filename: source/Data_homeless.xlsx, feature name: Homeless). The normalized homeless population of each state is the ratio between its homeless and total population.
- *Total cases (and deaths) of COVID-19* is the number of individuals tested positive and dead [37] (filename: source/Data_covid_total.xlsx, feature name: Total Cases and Total Death). The normalized infected/death is the ratio between the infected/death count to total population of the given state.
- *Infected score* and *death score* is obtained by rounding normalized total cases and deaths to discrete value between 0–6 (feature name: Infected Score, Death Score).
- *Death-to-Infected* is a feature measuring impact of death in terms of the difference between death and infected scores. It is calculated as *max*(*Death Score – Infected Score*, 0).
- *Lockdown type* is a feature capturing the type of lockdown (*shelter in place*: 1 and *stay at home*: 2) in a given state [37, 38] (filename: source/Data_lockdown.csv, feature name: Lockdown).
- *Day of lockdown* captures the difference in days between 1st January 2020 to the date of imposition of lockdown in a region [39] (filename: source/Data_lockdown.csv, feature name: Day Lockdown).
- *Population density* is the ratio between the population and area of a region [40] (filename: source/Data_population.csv, feature name: Population, Area, Population Density).
- *Traffic/activity of airport* measures the passenger traffic (also normalized by the total traffic across all the states of USA [41] (filename: source/Data_airport.xlsx, feature name: Busy airport score, Normalized busy airport).
- *Age groups* (0—80+) in brackets of 4 year (also normalized by total population) [40] (filename: source/Data_age.xlsx, feature name: age_to_, Norm_to_, e.g. age4to8); we later group them in brackets of 20 for the purposes of analysis.
- *Peak infected (and peak death)* measures the duration between first date of infection and date of daily infected (and death) peaks [40] (feature name: Peak Infected, Peak Death).
- *Testing* measures the number of individuals tested for COVID-19 (total number, before and after imposition of lockdown) [38, 42] (filename: source/Data_testing.xlsx, feature name: Testing, Pre-lockdown testing, Post-lockdown testing).
- *Pre- and post-infected and death count* measures the number of individuals infected and dead before and after lockdown dates (feature name: Testing, Pre-infected count, Pre-death count, Post-infected count, Post-death count).
- *Days between first infected and lockdown* date (feature name: First-Inf-Lockdown).

The above features, their abbreviations and summary statistics (i.e., mean, standard deviation, maximum and minimum) are enlisted in Table 1. Note that, for *gender* and *ethnicity* we report the fraction of the total state population falling in each category.

**Table 1:** Summary of features and their statistics (i.e., mean, standard deviation (dev.), maximum (max.) and minimum (min.)). The features in the order shown under “Feature name” are: GDP, inter-state distance based on lat-long coordinates, gender, ethnicity, quality of health care facility, number of homeless people, total infected and death, population density, airport passenger traffic, age group, days for infection and death to peak, number of people tested for COVID-19, days elapsed between first reported infection and the imposition of lockdown measures at a given state.

Feature name	Abbreviation	Mean	Dev.	Max	Min
Gross Domestic Product	*GDP*	412286.6	527087.5	3018337	34154
Distance	*d*	22.1	17.6	90.7	0.0
Gender	*Male*, *Female*	0.5	0.01	0.52	0.48
Ethnicity	*Wht*, *Blk*, *His*, *Asn*	0.24	0.28	0.93	0.0
Healthcare index	*health*	25.8	14.8	51.0	1.0
Homeless	*Home*	11963.48	21859.53	136826.0	946.0
Total Cases	*Inf*	32155.46	39521.26	168663.0	487.0
Total Death	*Dth*	1677.86	2428.85	11770.0	10.0
Population Density	*PD*	173.39	210.6	1035.64	1.12
Busy Airport Score	*Air*	375630.44	249207.97	1019704.0	100000.0
Age group	*age*	362738.87	439896.78	3125816.0	6853.0
Peak Infected	*P_Inf*	60.38	27.55	128.0	13.0
Peak Death	*P_dth*	58.88	23.7	112.0	14.0
Testing	*Test*	64353.04	24981.93	161172.0	31192.0
FirstInf-Lockdown	*Fst-Lock*	22.64	14.13	63.0	7.0

#### 2.1.2 Data from US states

The New York City (NYC) datasets (https://github.com/satunr/COVID-19/blob/master/US-COVID-Dataset/NYC_dist_mob.xlsx) show the inter-borough distance and mobility as well as COVID-19 infected (https://github.com/satunr/COVID-19/blob/master/US-COVID-Dataset/NYC-Inf.xlsx) and death counts (https://github.com/satunr/COVID-19/blob/master/US-COVID-Dataset/NYC-Dth.xlsx) for the 5 boroughs of NYC, namely, Manhattan, Queens, Brooklyn, Bronx and Staten Island.

- *Mobility data* (based on traffic volume counts collected by DOT for New York Metropolitan Transportation Council (NYMTC) [43]) shows the number of trips from one borough to another.
- COVID-19 data shows the number of *COVID-19 infected and death* counts for each borough [44].

#### 2.1.3 US infected and testing data

We acquire the daily infected and testing counts across US from January—July, 2020 [45]. This dataset is part of the COVID Tracking project that collect COVID-19 statistics on the numbers on tests, cases, hospitalizations, and patient outcomes from every US state and territory by voluntary public participation.

#### 2.1.4 Data preprocessing and normalization

We use the Scikit-learn library *KBinsDiscretizer* to group the continuous feature values into discrete values by creating balanced clusters using the quantile strategy [46].

#### 2.1.5 Supervised learning methods

Supervised machine learning algorithms learn a function that maps the input training data (i.e., features) to some output labels [47]. In this work, we consider the following supervised learning techniques. (Refer [48–54] for the details on these ML approaches.)

- *Support Vector Machine* (SVM) is used for classification and regression problems that maps the inputs to high-dimensional feature spaces. SVM operates on hyperplanes—decision boundaries that help classify the data points. The objective is to maximize the separation between the data points and the hyperplane. SVM is memory efficient and effective for datasets with fewer data samples [55].
- *Stochastic Gradient Descent* (SGD) is an iterative approach that fits the data to an objective function [56]. As the name suggests, it is a stochastic variant of the popular gradient descent (GD) optimization model [57]. In GD, the optimizer starts at a random point in the search space and reaches the lowest point of the function by traversing along the slope. Unlike GD that requires calculating the partial derivative for each feature at each data point, SGD achieves computational efficiency by computing derivatives on randomly chosen data points.
- *Nearest Centroid* (NC) is a simple classification model that represents each class by the centroid of its members. Subsequently, it assigns each data point to the cluster whose centroid is the closest to it. NC is particularly effective for non-convex classes and does not suffer from any additional dependencies on model parameters [58].
- *Decision Trees* (DTs) are a classification and regression technique that assigns target labels based on decision rules inferred from data features [59]. DT maintains the decision rules using a tree. A data point is assigned to a class by repeatedly comparing the tree root with the data point value to branch off to a new root.
- *Gaussian Naive Bayes* (NB) are a class of fast, probabilistic learning techniques that apply the Bayes’ theorem to assign labels to the data points [60].

While supervised ML approaches generally yield reliable prediction accuracy, they often suffer from overfitting or convergence issues [47, 61]. Each of the above approaches has its own advantages and disadvantages. SVM works well when the underlying distribution of the data is not known. However, it is prone to overfitting when the number of features is much greater than the number of samples. SGD needs low convergence time for a large dataset, but it may require to fit a number of hyperparameters. Conversely, DT involves almost no hyperparameters, but often entails slightly higher training time. Unlike DT, NB requires less training time but works on the implicit assumption that all the attributes are mutually independent. Finally, NC is a fast method but is not robust to outliers or missing data. In the context of our work, we intuit that the discriminatory feature(s) will yield a high accuracy irrespective of the underlying supervised ML algorithm used.

### 2.2 Metrics

- *Accuracy* function measures the fraction of matches between the predicted and actual labels in a multi-label classification, i.e., the ratio of correctly predicted observations to the total observations. It can be calculated as:
  $$\begin{array}{c} ACC=\frac{\text{TP}+\text{TN}}{\text{TP}+\text{TN}+\text{FP}+\text{FN}} \end{array}$$ (1)
  In the above equation, TP, TN, FP, FN denote true positive, true negative, false positive and false negative, respectively.
- *Extra trees classifier* is an estimator that fits randomized decision trees (called extra-trees) on data samples. The memory and computation overhead of this approach can be controlled by regulating the size of the extra trees. The nodes in the tree are split into sub-trees resulting in high accuracy (i.e., drop in impurity). Thus, feature *importance* is measured as total reduction in impurity affected by that feature [62].
- *Multiple regression* (MR) is a statistical tool to capture the linear relationship between the independent and the dependent variables *x* and *y* of a function *y* = *g*(*x*). In our context, MR generates a linear relationship $\hat{y}=\beta_{0}+\beta_{f_{1}}x_{f_{1}}+\beta_{f_{2}}x_{f_{2}}+\cdots +\epsilon$, where *b*_*fi*_ is the coefficient that captures the contribution of feature *f*_*i*_ towards the dependent variable *y*, while *β*_0_ and *ϵ* are the intercept and error terms, respectively.

### 2.3 Data correlation, standardization and error estimation

Given any pair of vectors *v* and $\hat{v}$ ($\left|v|=\right|\hat{v}|=n$), we apply the following standard statistical operations:

- *Mean centering* subtracts the mean *μ* from each element of a vector *v*, i.e., *v*′ = *v* − *μ*(*v*). This standardization adjusts the scales of magnitude by making the new mean 0 and helps compare data from varied sources or having different datatypes.
- *Mean squared error* (MSE) is calculated as $\frac{1}{n}\sum_{i=1}^{n}{\left(v_{i}-{\hat{v}}_{i}\right)}^{2}$.
- *Pearson Correlation Coefficient* (PCC) between *v* and $\hat{v}$ measures the strength of a linear association between two variables, where the value *PCC* = 1 is a perfect positive correlation and −1 is perfect negative correlation.
- *Positivity rate* *ρ* is the ratio between the number of individuals tested positive to the number of tests performed daily [63].

## 3 Results

This section is classified into the following three subsections: (1) and (2) identification and ranking of discriminatory factors, (3) effect on age and (4) feature influence on post-lockdown infection spread. The parameter values for the ML methods are summarized in Table 2. Unless otherwise stated, the *feature set* comprises *GDP*, *gender*, *ethnicity*, *health care*, *homeless*, *lockdown type*, *population density*, *airport activity*, and *age groups*, whereas the *output labels* consist of *infected and death scores* on a scale of 0–6.

**Table 2:** Values of parameters.

Method	Parameter
SVM	*kernel*: ‘RBF’; *regularization*: 1.0; *kernel function degree*: 3
SGD	*loss*: ‘hinge’; *penalty*: l2; *regularization* (*α*): 0.0001
NC	*distance metric*: ‘euclidean’
DT	*split criterion*: ‘gini’; *split strategy*: ‘best’; *maximum tree depth* (*max*_*depth*): ‘None’
NB	*largest feature variance*: 10^−9^
Extra trees	*number of trees*: 100; *split criterion*: ‘gini’; *maximum tree depth* (*max*_*depth*): ‘None’
Regression	*fit_intercept*: ‘True’; *normalize feature* (*normalize*): ‘False’
KBinsDiscretizer	’Number of bins’: 5

### 3.1 Identification of discriminatory factors

**Fig 1:**
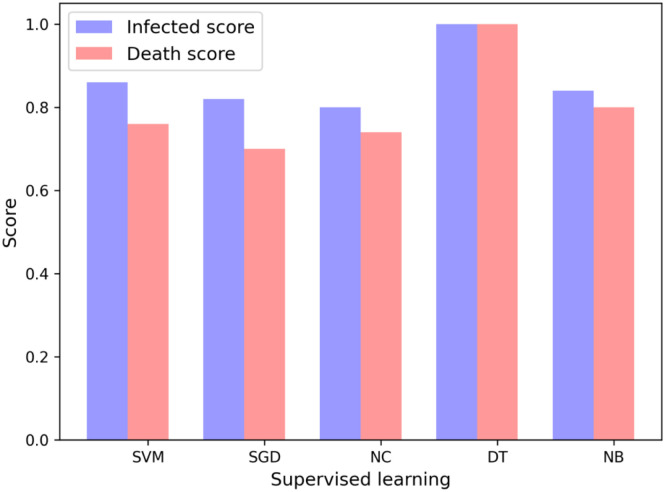
Accuracy scores of the 5-tuple of features for the output variables of infected and death scores for different supervised learning techniques.

We apply supervised machine learning (ML) approaches to identify the key factors affecting COVID-19 infected and death counts. For each supervised ML technique, we perform an exhaustive search of all possible combinations of any 5 features and identify the feature subset(s) with the highest accuracy (discussed in Sec. 2.2) as the most important features. Fig 1 shows the scores for different supervised methods. Although proposing a machine learning algorithm that works best on COVID-19 data is not the purpose of this study, it is worth reporting that decision tree classifier (DT) slightly outperforms the other algorithms for both cases of infected and death scores.

*Feature combinations*: For each supervised learning technique, several 5-tuples of features may yield the same accuracy score. For instance, suppose that (*home*, *dth*, *male*, *test*, *Inf*) and (*home*, *dth*, *air*, 40_44, *Inf*) yield the same accuracy. (Recall that 40_44 signifies the feature population in age group 40 to 44.) Consequently, one feature can participate in several combinations. For any supervised learning method *ρ*, let *C* = {*c*_1_, *c*_2_, ⋯} be a list of feature combinations with the highest scores, where *c*_*i*_ is a 5-tuple of features. We attempt to gauge the importance of a feature *f*_*i*_, *I*(*f*_*i*_), by the fraction of combinations in *C* it participates in, i.e., $I_{\rho }\left(f_{i}\right)=\frac{|c_{i}:f_{i}\in c_{i}\&c_{i}\in C|}{\left|C\right|}$.

**Fig 2:**
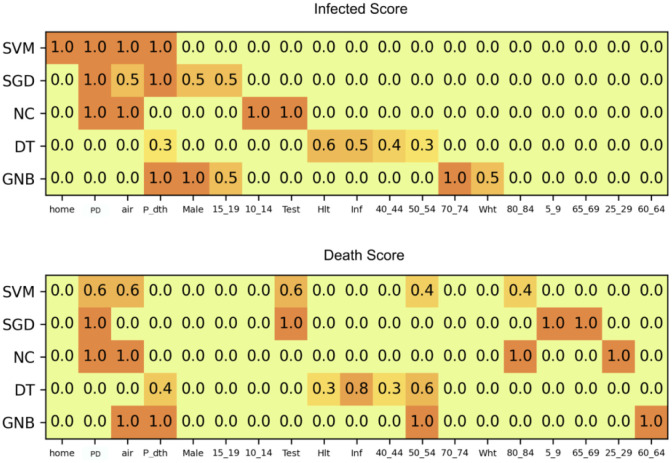
Participation of features in 5-tuples of key feature combinations for infected score (top) and death score (bottom). Refer Table 1 for the feature abbreviations.

We create a pool of all features participating in at least one combination for output labels of infected and death scores. Fig 2 shows a heatmap of the importance *I* for all such features against each supervised technique. For infected score as output label (top figure), *homeless* (home), *population density* (PD), *airport activity* (air), *testing* (test), *white* (wht), etc. have the highest *I*. For death score as output label, PD, air, test and age groups above 50 years (age50_54 and age80_84) exhibit the highest importance.

### 3.2 Ranking of discriminatory features

**Fig 3:**
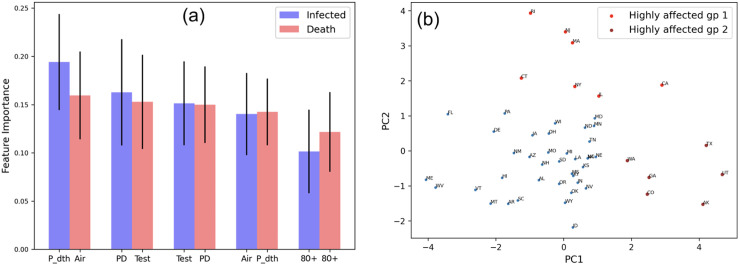
Feature importance: (a) 5 discriminatory features along with their importance scores and standard deviation (in the decreasing order) affecting infected and death scores based on randomized decision trees; (b) principal component analysis on the 5 features showing that the most highly COVID-19 affected states form two groups: (1) early epicenters colored red and (2) states experiencing strong second wave or peaking late w.r.t. infection and death counts (colored brown).

We apply the extra trees classifier to generate the impurity-based rank for the features (discussed in Sec. 2.2). Fig 3a shows the top 5 important features corresponding to the infected and death scores, respectively. It is interesting that for both cases, the same set of features, namely, *population density*, *days to peak*, *airport traffic*, *testing* and *high age groups*, are identified. Also note that the same features exhibit a very high participation in the 5-feature combinations shown in Fig 2. Next, as a validation exercise, we apply dimension reduction on the top 5 features (selected by supervised ML approaches) for the 50 US states. Fig 3b shows the PCA plots where the most highly COVID-19 affected US states form two groups (that stand out of the largest cluster colored blue): states (1) that were the early epicenters of pandemic (colored red) such as California, New Jersey, New York, Rhode Island, Illinois and Connecticut and (2) showing a strong second wave or peaking late in infection and death counts (colored brown) such as Texas, Arkansas, Washington, Georgia, Colorado and Utah [64–69].

### 3.3 Effect on age

**Fig 4:**
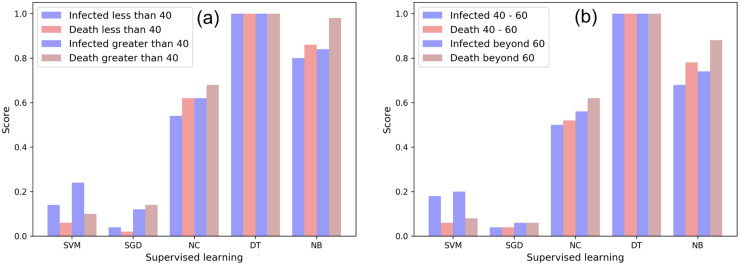
Effect of COVID-19 infected and death score on age: Comparison of accuracy scores for feature set of (a) age (≤40, >40) and (b) age (40–60, beyond 60).

We discussed in Sec. 2.1, that our initial dataset groups ages into brackets of 4 (0–4, 4–8, and so on). Our results from supervised learning (Sec. 3.1) and extra trees (Sec. 3.2) suggest that high age groups are important factors affecting the infected and death scores of COVID-19. To understand the effect of COVID-19 infected and death scores on low and high age groups, we create two feature sets for population of age ≤40 and >40. Fig 4a shows that for both cases of infected and death, the accuracy (*ACC*) is higher for higher age groups. We explore this by repeating the above experiment, this time, with a feature set of groups 40–60 and >60. Fig 4b depicts that *ACC* for age group 60+ is marginally higher, suggesting that the elderly are amongst the most vulnerable, however the difference in mortality rates in this case was not statistically significant.

### 3.4 Feature influence on post-lockdown infection spread

We carry out a study to identify the pre-lockdown factors of any region (US states in our case) that contribute to the overall post-lockdown infection and death numbers. We partition the total infected and death numbers for each state into pre- and post-lockdown infected and death counts. We then create a feature set consisting of *population density*, *airport business*, *pre-lockdown infected*, *pre-lockdown death*, *days between first infected to lockdown* and *age group above 80*. The features represent the set of observable factors for the administrative and health bodies and were already shown to possess high feature significance in the previous section. The output labels are the *post-lockdown infected* and *post-lockdown death* numbers. We perform the following experiments:

#### 3.4.1 Identification of discriminating features

**Fig 5:**
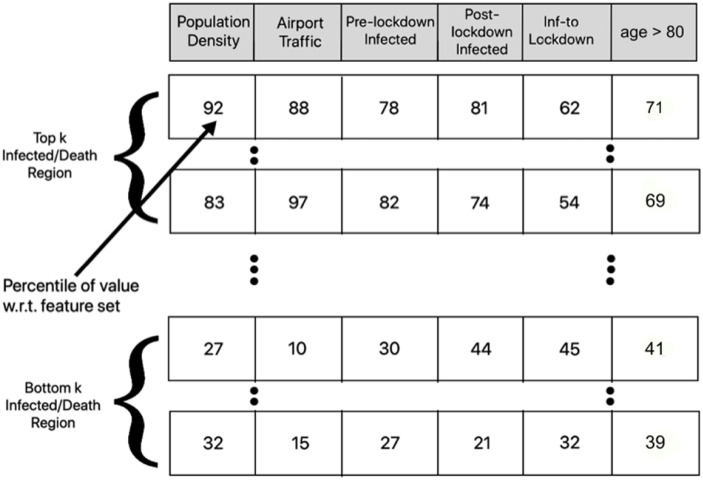
Preprocessing to study the variation in feature values for the top and bottom *k* US states on the basis of COVID-19 infected and death scores.

**Fig 6:**
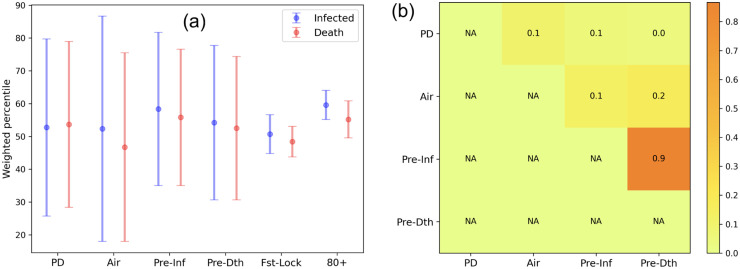
Identification of discriminating features: (a) maximum difference in weighted average percentile for top and bottom *k* COVID-19 affected US; (b) heatmap showing the pairwise Pearson correlation correlation between discriminating features.

We carry out a simple preprocessing step to convert each feature entry to percentile (with respect to the feature vector) and rank the US states in the decreasing order of infected and death scores (Fig 5). We calculate the weighted average percentile of features for the top and bottom *k* = 10 US states using the formula $\frac{1}{\sum_{i=1}^{r}\rho \left(f_{i}\right)}\sum_{i=1}^{k}p\left(f_{i}\right)\times \left(r-\rho \left(f_{i}\right)\right)$, where *p*(*f*_*i*_) and *ρ*(*f*_*i*_) are the percentile and rank of the *i*^*th*^ feature value, while *r* is the number of US states (equal to maximum rank). We intuit that the feature exhibiting the maximum difference in weighted average percentile for top and bottom *k* COVID-19 affected US states are the discriminating ones. Fig 6a shows the percentile difference suggesting that airport and population density are the most significant, while days between first infected to lockdown and age group of 80+ are the least discriminating.

#### 3.4.2 Feature weights based on multiple regression

We apply multiple regression (MR) (see Sec. 2.2) to measure the weightage of each of the above features in the observed *post-lockdown infected* (Post_Inf) and *post-death numbers* (Post_Dth). We eliminate the days between *first infected to lockdown* (Fst-Lock) and age group 80+, which are the least discriminating features from the percentile analysis (see Fig 6a). As a prerequisite for MR, we need to eliminate features that are mutually correlated. Fig 6b shows that *Pre-inf* and *Pre-dth* are highly correlated, and hence we run two separate batches of MR: (1) *population density*, *airport business*, *pre-lockdown infected* and (2) *population density*, *airport business*, *pre-lockdown death*.

#### 3.4.3 Effect of testing and lockdown on infection spread

**Fig 7:**
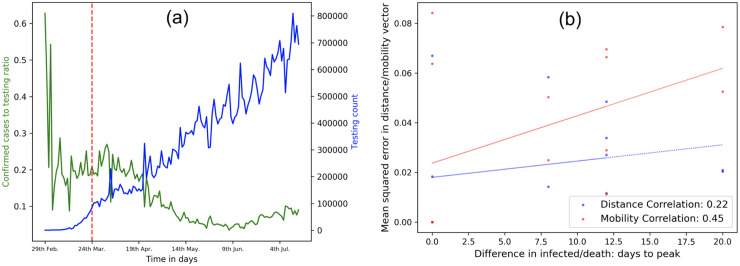
Role of mobility and testing on spread: (a) the effect of testing and lockdown on infection spread: Testing rate (blue line) increases steadily over time and confirmed cases to testing ratio drops post lockdown due to reduced contact; (b) correlation between mobility (or distance) and days for infected to peak in neighboring NYC boroughs.

We explore the effect of testing and lockdown on infection spread. We utilize positivity ratio *ρ* (defined in Sec. 2.3) to gauge how widespread the infection spread is [63]. We acquire the daily infected and testing count in US (see Sec. 2.1.3) and plot the mean daily *ρ* across all states over the period of February—July 2020. Fig 7a shows that the testing increased over a period time, while the positivity ratio dropped post lockdown (shown in red dotted line). While, testing (and, by extension, positivity ratio) is an effective epidemiological indicator, it cannot curb infection spread by itself. However, Fig 7a shows that the *ρ* has dropped approximately three weeks into the lockdown, suggesting that the latter had an impact on curbing spread by minimizing social contact. Table 3 shows that pre-infected and pre-death with high coefficients contribute highly towards the post-lockdown infected and death numbers, followed by population density and airport traffic. This finding is further supported by the p values reported for the respective features. Note that the *R*^2^ scores for all the four cases are >0.8, suggesting that the output features capture a high proportion of the variance in the input features. Overall, pre-infected count has higher coefficient and *R*^2^ score and emerges as a marginally better discriminating feature of post-lockdown effects than the pre-death count.

**Table 3:** Multiple linear regression table with *R*^2^, coefficient and p value for input features (*population density, normalized busy airport, pre-infected count, pre-death count*) and observed factors (*post-infected count* and *post-death count*).

*Input Feature*	*Output Feature*	*R*^2^	*Coeff*.	*p value*
Constant	Post-Inf	0.92	-0.92	0.068
PD			0.17	0
Air			0.19	0
Pre-Inf			0.81	0
Constant	Post-Dth	0.94	-0.68	0.106
PD			0.17	0
Air			0.06	0.005
Pre-Inf			0.91	0
Constant	Post-Inf	0.83	-1.37	0.074
PD			0.20	0
Air			0.18	0
Pre-Dth			0.67	0
Constant	Post-Dth	0.82	-1.17	0.116
PD			0.20	0
Air			0.05	0.213
Pre-Dth			0.76	0

## 4 Discussions

In Sec. 3.2, we perform PCA on the feature set of the key factors to show that states with high infection and death numbers stand out of the cluster of other states. These states include some erstwhile hotspots forming group 1 (such as New York City, New Jersey, Massachusetts, Connecticut, Rhode Island) as well as states experiencing a steady infection and death count and also a strong second wave forming group 2 (such as Texas, Washington, California, Georgia, Arkansas, Utah and Colorado) (Fig 3b). In the PCA analysis, PC1 and PC2 account for 41% and 21% variance, respectively. We explore how each feature influences each component to show that PC1 is driven by factors such as airport activity and high age groups (70 and beyond), while PC2 is dominated by population density, airport, age (80+) and testing. Notice in Fig 3b, though both groups 1 and 2 exhibit high spread across PC1, group 2 forms a slightly denser cluster than group 1, implying that it exhibits an even mix of PC1 and PC2 features. We intuit that the early peaking in infection in group 1 states is due to high road and airport mobility leading to high mixing and infection spread that is manifested in the elderly population. Group 2 shows enduring infection spread due to high population density and testing, in addition to airport activity and populations with higher age group.

We study how demographics affect COVID-19 numbers to show that states with higher age groups (particularly 60 and beyond) numbers are the most vulnerable. Finally, we split the infected and death numbers on the pre- and post-lockdown epochs and apply multiple linear regression to show that pre-lockdown infected and death, population density and airport contribute highly to the post-lockdown numbers. This analysis can be particularly effective in pinpointing the most vulnerable states and recommending lockdown policies on starting dates and duration to curb pandemic spread. Note that our present study pertains to the identification of the discriminatory features with respect to the date of lockdown. There exists several unanswered questions regarding the impact of length, scheduling strategies, lockdown types and extent of lockdowns on pandemic spread that need to be answered. Such an analysis requires a richer feature set as well as a sound understanding of the dynamics of infection spread in terms of healthcare, distance, mobility, etc. As a preliminary study, we first explore whether there is any relationship between the health care index (*Health*) of a US state and the number of transitions from infected to death (*Dth*/*Inf*) in this state. The Pearson’s correlation coefficient (see Sec. 2.3) between the two factors is 0.11, suggesting that the overall mortality numbers is largely unrelated to the healthcare facility and may solely depend on the infected individual’s attributes, such as age, comorbidities, infection severity, etc.

Second, since proximity plays a role in infection spread, neighboring regions should peak at nearly the same time. We posit that mobility may play an even greater role in the spread, than a static measure like distance between a pair of regions. In the absence of a inter-state mobility dataset, we create two feature sets for the NYC boroughs dataset (see Sec. 2.1): (1) inter-borough distance and (2) inter-borough mobility. Each borough *b* has a distance and mobility vector *D*_*b*_ = {*d*_*b*1_, *d*_*b*2_⋯} and *M*_*b*_ = {*m*_*b*1_, *m*_*b*2_⋯} where *d*_*bi*_ and *m*_*bi*_ are the probabilistic measure of distance and mobility between a borough *b* with borough *i*. We calculate the correlation of the mean squared error (see Sec. 2.3) of the distance/mobility vectors of any pair of boroughs *b*_1_ and *b*_2_ against the absolute difference of their peak to infected or peak-to-death features. Fig 7b suggests that mobility yields a higher correlation (0.44) than distance (0.22) suggesting that mobility is a slightly more informative feature to analyze infection spread.

We are currently working towards broadening the scope of this study in different directions. First, this work attempted to apply ML analysis on a wide range of features, making the the states of United States the ideal choice, specifically from the standpoint of data availability. In future we would like to extend this work by running these experiments on epidemiological, demographic and economic data of different countries. It would be interesting to report the variation in the discriminatory features identified for different countries. Second, we identify population density, testing, airport activity and pre-lockdown infected count as key features driving the post-lockdown infection and death numbers. We plan to utilize these findings to design policies on the timing, duration and stringency of lockdown for future pandemics. Third, all the input features discussed in this work are static or time invariant. It is imperative to analyze the evolution of dynamic features (such as GDP and unemployment rates) from the pre-COVID to the post-COVID timelines to uncover the long-term economic effects of COVID-19.

## 5 Conclusions

Machine learning is emerging as an important tool to predict the dynamics of spread of COVID-19 and identify the key factors driving infection and mortality rates. While existing works study the effects of gender, race, age, testing, social contact and distancing separately, we present an unified analysis of the demographic, economic, and epidemiological, ethnic and health indicators for infection and mortality rates from COVID-19. We curate a dataset of US states comprising features (from varying sources discussed in Sec. 2.1) that may potentially impact infection and death rates of COVID-19. We run several supervised machine learning techniques to identify and rank the key factors correlating with infection and fatality counts. *Population density*, *testing rate*, *airport traffic*, *high age groups* emerge as significant, while *ethnicity*, *gender*, *healthcare index*, *homeless* and *GDP* have little or no impact on pandemic spread and mortality.

## Supporting information

S1 File (CLS)

S2 File (BST)

S3 File (STY)

S4 File (BST)
