# Supplementary material for: Factors affecting COVID-19 infected and death rates inform lockdown-related policymaking
Source: PLoS One. 2020 Oct 23;15(10):e0241165. doi: 10.1371/journal.pone.0241165 (PMC7584177; doi:10.1371/journal.pone.0241165)
Supplement: S4 File — (BST) [file pone.0241165.s004.bst]

%%%% %%%%%% This `plos2015.bst' file is intended for use in PLOS submissions to %% correctly compile the bibliography according to the PLOS manuscript %% guidelines updated January 2015.%% %%%% This is derived from the `vancouver.bst' bibliographic style file (for %% LaTeX/BibTeX), generated with the docstrip utility and modified manually%% to meet the%% ``Uniform Requirements for Manuscripts Submitted to Biomedical Journals''%% as published in N Engl J Med 1997;336:309-315.%% (also known as the Vancouver style)%% This specification may be found on the web page of the%% International Committe of Medical Journal Editors:%%%% http://www.icmje.org%%%%-------------------------------------------------------------------%%%% Copyright 2004  Folkert van der Beek%%%% This work may be distributed and/or modified under the%% conditions of the LaTeX Project Public License, either version 1.3%% of this license or (at your option) any later version.%% The latest version of this license is in%%   http://www.latex-project.org/lppl.txt%% and version 1.3 or later is part of all distributions of LaTeX%% version 2005/12/01 or later.%%%% This work has the LPPL maintenance status `maintained'.%% %% The Current Maintainer of this work is Folkert van der Beek.%%%% Complaints, suggestions and comments may be sent to%%%% Folkert van der Beek <folkertvanderbeek at gmail dot com>%%%%-------------------------------------------------------------------%%%% This bibliography style file is intended for texts in ENGLISH%% This is a numerical citation style, and as such is standard LaTeX.%% It requires no extra package to interface to the main text.%% The form of the \bibitem entries is%%   \bibitem{key}...%% Usage of \cite is as follows:%%   \cite{key} ==>>          [#]%%   \cite[chap. 2]{key} ==>> [#, chap. 2]%% where # is a number determined by the ordering in the reference list.%% The order in the reference list is that by which the works were originally%%   cited in the text, or that in the database. %%% To change the reference numbering system from [1] to 1,%% put the following code in the preamble:%% \makeatletter % Reference list option change%% \renewcommand\@biblabel[1]{#1} % from [1] to 1%% \makeatother %%%%%---------------------------------------------------------------------%% List of all possible fieldsENTRY  { address    assignee     % for patents    author    booktitle    % for articles in books    chapter      % for incollection, esp. internet documents    cartographer % for maps    day    edition    editor    howpublished    institution  % for technical reports    inventor     % for patents    journal    key    month    note    number    organization    pages    part    publisher    school    series    title    type    volume    word    year    eprint % urlbst    doi % urlbst    url % urlbst    lastchecked % urlbst    updated % urlbst  }  {}  { label }%% Declaration of integer variablesINTEGERS { output.state before.all mid.sentence after.sentence after.block }STRINGS { urlintro eprinturl eprintprefix doiprefix doiurl } % urlbst...INTEGERS { hrefform addeprints adddoiresolver }% Following constants may be adjusted by hand, if desiredFUNCTION {init.config.constants}{  "Available from: " 'urlintro := % prefix before URL  "http://arxiv.org/abs/" 'eprinturl := % prefix to make URL from eprint ref  "arXiv:" 'eprintprefix := % text prefix printed before eprint ref  "http://dx.doi.org/" 'doiurl := % prefix to make URL from DOI  "doi:" 'doiprefix := % text prefix printed before DOI ref  #0 'addeprints := % 0=no eprints; 1=include eprints  #0 'adddoiresolver := % 0=no DOI resolver; 1=include it  #0 'hrefform := % 0=no crossrefs; 1=hypertex xrefs; 2=hyperref refs}INTEGERS {   bracket.state  outside.brackets  open.brackets  within.brackets  close.brackets}% ...urlbst to hereFUNCTION {init.state.consts}{ #0 'outside.brackets := % urlbst  #1 'open.brackets :=  #2 'within.brackets :=  #3 'close.brackets :=  #0 'before.all :=  #1 'mid.sentence :=  #2 'after.sentence :=  #3 'after.block :=}%% Declaration of string variablesSTRINGS { s t}% urlbstFUNCTION {output.nonnull.original}{ 's :=  output.state mid.sentence =    { ". " * write$ }    { output.state after.block =        { add.period$ write$          newline$          "\newblock " write$        }        { output.state before.all =            'write$            { add.period$ " " * write$ }          if$        }      if$      mid.sentence 'output.state :=    }  if$  s}% urlbst...FUNCTION {output.nonnull}{ % Save the thing we've been asked to output  's :=  % If the bracket-state is close.brackets, then add a close-bracket to  % what is currently at the top of the stack, and set bracket.state  % to outside.brackets  bracket.state close.brackets =    { "]" *      outside.brackets 'bracket.state :=    }    'skip$  if$  bracket.state outside.brackets =    { % We're outside all brackets -- this is the normal situation.      % Write out what's currently at the top of the stack, using the      % original output.nonnull function.      s      output.nonnull.original    }    { % Still in brackets.  Add open-bracket or (continuation) comma, add the      % new text (in s) to the top of the stack, and move to the close-brackets      % state, ready for next time (unless inbrackets resets it).  If we come      % into this branch, then output.state is carefully undisturbed.      bracket.state open.brackets =        { " [" * }        { ", " * } % bracket.state will be within.brackets      if$       s *       close.brackets 'bracket.state :=    }  if$}% Call this function just before adding something which should be presented in % brackets.  bracket.state is handled specially within output.nonnull.FUNCTION {inbrackets}{ bracket.state close.brackets =    { within.brackets 'bracket.state := } % reset the state: not open nor closed    { open.brackets 'bracket.state := }  if$}FUNCTION {format.lastchecked}{ lastchecked empty$    { "" }    { updated empty$      { inbrackets "cited " lastchecked * }      { inbrackets "updated " updated * "; cited " * lastchecked * }    if$    }  if$}% ...urlbst to hereFUNCTION {output}{ duplicate$ empty$    'pop$    'output.nonnull  if$}FUNCTION {output.check}{ 't :=  duplicate$ empty$    { pop$ "empty " t * " in " * cite$ * warning$ }    'output.nonnull  if$}FUNCTION {fin.entry}{   bracket.state close.brackets = % urlbst    { "]" * }    'skip$  if$   add.period$  write$  newline$}FUNCTION {new.block}{ output.state before.all =    'skip$    { after.block 'output.state := }  if$}FUNCTION {new.sentence}{ output.state after.block =    'skip$    { output.state before.all =        'skip$        { after.sentence 'output.state := }      if$    }  if$}FUNCTION {add.blank}{  " " * before.all 'output.state :=}FUNCTION {no.blank.or.punct}{  "" * before.all 'output.state :=}FUNCTION {add.semicolon}{  ";" *  no.blank.or.punct}FUNCTION {date.block}{  "." *  no.blank.or.punct}%%%%%%%%%%%%%%%%%%%%%%%%%%%%%%%%%%%%%%%%%%%%%%%%%%%%%%%%%%%%%%            LOGICAL `NOT', `AND', AND `OR'                 %%%%%%%%%%%%%%%%%%%%%%%%%%%%%%%%%%%%%%%%%%%%%%%%%%%%%%%%%%%%%%%%%%%%%%%%%%%%%%%%%%%%%%%%%%%%%%%%%%%%%%%%%%%%%%%%%%%%%%%%%%%% Logical 'not':% If the first element on the stack is A then this function% does the following:%     push { #0 }%     push { #1 }% So now the first 3 elements of the stack are%     { #1 } { #0 } A% The first 3 are popped and subjected to 'if':% If A > 0 then { #0 } is executed, else { #1 } is executed:%     if A > 0%     then 0%     else 1% So consider integers as logicals, where 1 = true and 0 = false,% then this does%     (if A then false else true)% which is a logical 'not'.FUNCTION {not}{   { #0 }    { #1 }  if$}%%%%%%%%%%%%%%%%%%%%%%%%%%%%%%%%%%%%%%%%%%%%%%%%%%%%%%%%%%%%%% Logical 'and':% If the first 2 elements on the stack are A B% then this function does the following:%     push 'skip$%     push { pop$ #0 }% So now first 4 elements are%     { pop$ #0 } 'skip$ A B% The first 3 are popped and subjected to 'if' (B is on top of% the stack):% If A > 0 then 'skip$ is executed, else { pop$ #0 } is executed:%     if A > 0%     then (B stays on top of stack)%     else (B is popped and #0 is pushed)% So consider integers as logicals, where 1 = true and 0 = false,% then this does%     (if A then B else false)% which is a logical 'and'.FUNCTION {and}{   'skip$    { pop$ #0 }  if$}%%%%%%%%%%%%%%%%%%%%%%%%%%%%%%%%%%%%%%%%%%%%%%%%%%%%%%%%%%%%%% Logical 'or':% If the first 2 elements on the stack are A B% then this function does the following:%     push { pop$ #1 }%     push 'skip$% So now first 4 elements are%     'skip$ { pop$ #1 } A B% The first 3 are popped and subjected to 'if' (B is on top of% the stack):% If A > 0 then { pop$ #1 } is executed, else 'skip$ is executed:%     if A > 0%     then (B is popped and #1 is pushed)%     else (B stays on top of stack)% So consider integers as logicals, where 1 = true and 0 = false,% then this does%     (if A then true else B)% which is a logical 'or'.FUNCTION {or}{   { pop$ #1 }    'skip$  if$}%%%%%%%%%%%%%%%%%%%%%%%%%%%%%%%%%%%%%%%%%%%%%%%%%%%%%%%%%%%%%%  GENERAL PURPOSE FUNCTIONS FOR FORMATTING                 %%%%%%%%%%%%%%%%%%%%%%%%%%%%%%%%%%%%%%%%%%%%%%%%%%%%%%%%%%%%%%%%%%%%%%%%%%%%%%%%%%%%%%%%%%%%%%%%%%%%%%%%%%%%%%%%%%%%%%%%%%%% issues warning if field is empty% call with%    "field"  field  warning.if.empty% Note that the first field must be between quotes% because it is the fieldname for use in the warning message.%FUNCTION {warning.if.empty}{ empty$    { "No "  swap$ * " in " * cite$ * warning$ }    { pop$ }  if$}%%%%%%%%%%%%%%%%%%%%%%%%%%%%%%%%%%%%%%%%%%%%%%%%%%%%%%%%%%%%%    %    % encloses string in pre- and postfix string    % call with    %    prefix postfix  S  enclose.check    % delivers empty string if S empty    %FUNCTION {enclose.check}{ duplicate$ empty$    { pop$ pop$ pop$      ""    }    { swap$ * * }  if$}%%%%%%%%%%%%%%%%%%%%%%%%%%%%%%%%%%%%%%%%%%%%%%%%%%%%%%%%%%%%%%% emphasizes top of stack% call with%    string" emphasize.check%FUNCTION {emphasize.check}{ "\Bem{" swap$  "}"     swap$  enclose.check}%%%%%%%%%%%%%%%%%%%%%%%%%%%%%%%%%%%%%%%%%%%%%%%%%%%%%%%%%%%%%    %    % brackets top of stack    % call with    %     "string" bracket.check    %FUNCTION {bracket.check}{ "[" swap$  "]" swap$  enclose.check}%%%%%%%%%%%%%%%%%%%%%%%%%%%%%%%%%%%%%%%%%%%%%%%%%%%%%%%%%%%%%    %    % parenthesizes top of stack    % call with    %     "string" parenthesize    %FUNCTION {parenthesize.check}{ "(" swap$  ")" swap$  enclose.check}STRINGS {z}FUNCTION {remove.dots}{ 'z :=	% expects string on top of the stack, pops the string and assigns it to variable z  "" % push empty string  { z empty$ not } % returns 0 if variable z is empty  { z #1 #1 substring$ % push the first character of variable z    z #2 global.max$ substring$ 'z := % assigns the 2nd to last character of variable z to variable z    duplicate$ "\" = % pushes 1 if the last character is "\", otherwise 0    { * % concatenates the last 2 literals      z #1 #1 substring$ % push the first character of variable z      z #2 global.max$ substring$ 'z := % assigns the 2nd to last character of variable z to variable z      * % concatenates the last 2 literals, i.e. every character, even a dot, following a "\" will be printed    }    { duplicate$ "." = % pushes 1 if the last character is ".", otherwise 0      'pop$ %  pushes the pop$ function       { * } % concatenates the last 2 literals    if$ % pops the last character if it is a dot, otherwise concatenates it with the string on top of the stack    }    if$  }  while$}INTEGERS {l}FUNCTION{string.length}{  #1 'l :=  { duplicate$ duplicate$ #1 l substring$ = not }    { l #1 + 'l := }  while$  pop$ l}STRINGS {replace find text}INTEGERS {find_length}FUNCTION {find.replace}{   'replace :=  'find :=  'text :=  find string.length 'find_length :=  ""    { text empty$ not }    { text #1 find_length substring$ find =      {        replace *        text #1 find_length + global.max$ substring$ 'text :=      }      { text #1 #1 substring$ *        text #2  global.max$ substring$ 'text :=      }    if$    }  while$}FUNCTION {new.block.checka}{ empty$    'skip$    'new.block  if$}FUNCTION {new.block.checkb}{ empty$  swap$ empty$  and    'skip$    'new.block  if$}FUNCTION {new.sentence.checka}{ empty$    'skip$    'new.sentence  if$}FUNCTION {new.sentence.checkb}{ empty$  swap$ empty$  and    'skip$    'new.sentence  if$}FUNCTION {field.or.null}{ duplicate$ empty$    { pop$ "" }    'skip$  if$}FUNCTION {emphasize}{ skip$ }FUNCTION {tie.or.space.prefix}{ duplicate$ text.length$ #3 <    { "~" }    { " " }  if$  swap$}FUNCTION {capitalize}{ "u" change.case$ "t" change.case$ }FUNCTION {space.word}{ " " swap$ * " " * } % Here are the language-specific definitions for explicit words. % Each function has a name bbl.xxx where xxx is the English word. % The language selected here is ENGLISHFUNCTION {bbl.and}{ "and"}FUNCTION {bbl.etal}{ "et~al." }FUNCTION {bbl.editors}{ "editors" }FUNCTION {bbl.editor}{ "editor" }FUNCTION {bbl.cartographers}{ "cartographers" }FUNCTION {bbl.cartographer}{ "cartographer" }FUNCTION {bbl.inventors}{ "inventors" }FUNCTION {bbl.inventor}{ "inventor" }FUNCTION {bbl.assignees}{ "assignees" }FUNCTION {bbl.assignee}{ "assignee" }FUNCTION {bbl.edby}{ "edited by" }FUNCTION {bbl.edition}{ "ed." }FUNCTION {bbl.volume}{ "vol." }FUNCTION {bbl.of}{ "of" }FUNCTION {bbl.number}{ "no." }FUNCTION {bbl.nr}{ "no." }FUNCTION {bbl.in}{ "in" }FUNCTION {bbl.pages}{ "p." }FUNCTION {bbl.page}{ "p." }FUNCTION {bbl.chapter}{ "chap." }FUNCTION {bbl.techrep}{ "Tech. Rep." }FUNCTION {bbl.mthesis}{ "Master's thesis" }FUNCTION {bbl.phdthesis}{ "Ph.D. thesis" }FUNCTION {bbl.first}{ "1st" }FUNCTION {bbl.second}{ "2nd" }FUNCTION {bbl.third}{ "3rd" }FUNCTION {bbl.fourth}{ "4th" }FUNCTION {bbl.fifth}{ "5th" }FUNCTION {bbl.st}{ "st" }FUNCTION {bbl.nd}{ "nd" }FUNCTION {bbl.rd}{ "rd" }FUNCTION {bbl.th}{ "th" }MACRO {jan} {"Jan."}MACRO {feb} {"Feb."}MACRO {mar} {"Mar."}MACRO {apr} {"Apr."}MACRO {may} {"May"}MACRO {jun} {"Jun."}MACRO {jul} {"Jul."}MACRO {aug} {"Aug."}MACRO {sep} {"Sep."}MACRO {oct} {"Oct."}MACRO {nov} {"Nov."}MACRO {dec} {"Dec."}FUNCTION {eng.ord}{ duplicate$ "1" swap$ *  #-2 #1 substring$ "1" =     { bbl.th * }     { duplicate$ #-1 #1 substring$       duplicate$ "1" =         { pop$ bbl.st * }         { duplicate$ "2" =             { pop$ bbl.nd * }             { "3" =                 { bbl.rd * }                 { bbl.th * }               if$             }           if$          }       if$     }   if$}FUNCTION {bibinfo.check}{ swap$  duplicate$ missing$    {      pop$ pop$      ""    }    { duplicate$ empty$        {          swap$ pop$        }        { swap$          pop$        }      if$    }  if$}FUNCTION {bibinfo.warn}{ swap$  duplicate$ missing$    {      swap$ "missing " swap$ * " in " * cite$ * warning$ pop$      ""    }    { duplicate$ empty$        {          swap$ "empty " swap$ * " in " * cite$ * warning$        }        { swap$          pop$        }      if$    }  if$}STRINGS  { bibinfo}INTEGERS { nameptr namesleft numnames }FUNCTION {format.names}{ 'bibinfo :=  duplicate$ empty$ 'skip$ {  "." ". " find.replace 's :=  "" 't :=  #1 'nameptr :=  s num.names$ 'numnames :=  numnames 'namesleft :=    { namesleft #0 > }    { s nameptr      "{vv~}{ll}{ f{}}{ jj}"      format.name$      remove.dots      bibinfo bibinfo.check      't :=      nameptr #1 >        {          nameptr #6          #1 + =          numnames #6          > and            { "others" 't :=              #1 'namesleft := }            'skip$          if$          namesleft #1 >            { ", " * t * }            {              "," *              s nameptr "{ll}" format.name$ duplicate$ "others" =                { 't := }                { pop$ }              if$              t "others" =                {                  " " * bbl.etal *                }                { " " * t * }              if$            }          if$        }        't      if$      nameptr #1 + 'nameptr :=      namesleft #1 - 'namesleft :=    }  while$  } if$}FUNCTION {format.names.org}{ 'bibinfo :=  duplicate$ empty$ 'skip$ {  's :=  "" 't :=  #1 'nameptr :=  s num.names$ 'numnames :=  numnames 'namesleft :=    { namesleft #0 > }    { s nameptr      "{ff~}{vv~}{ll}"      format.name$      bibinfo bibinfo.check      't :=      nameptr #1 >        {          namesleft #1 >            { "; " * t * }            {              ";" *              s nameptr "{ll}" format.name$ duplicate$ "others" =                { 't := }                { pop$ }              if$              t "others" =                {                  " " * bbl.etal *                }                { " " * t * }              if$            }          if$        }        't      if$      nameptr #1 + 'nameptr :=      namesleft #1 - 'namesleft :=    }  while$  } if$}FUNCTION {format.names.ed}{  format.names}FUNCTION {format.authors}{   author "author" format.names  %%"." " " "author" find.replace format.names}FUNCTION {format.organizations}{ organization "organization" format.names.org}FUNCTION {get.bbl.editor}{ editor num.names$ #1 > 'bbl.editors 'bbl.editor if$ }FUNCTION {get.bbl.cartographer}{ cartographer num.names$ #1 > 'bbl.cartographers 'bbl.cartographer if$ }FUNCTION {get.bbl.inventor}{ inventor num.names$ #1 > 'bbl.inventors 'bbl.inventor if$ }FUNCTION {get.bbl.assignee}{ assignee num.names$ #1 > 'bbl.assignees 'bbl.assignee if$ }FUNCTION {format.editors}{ editor "editor" format.names duplicate$ empty$ 'skip$    {      "," *      " " *      get.bbl.editor      *    }  if$}FUNCTION {format.assignees}{ assignee "assignee" format.names.org duplicate$ empty$ 'skip$    {      "," *      " " *      get.bbl.assignee      *    }  if$}FUNCTION {format.cartographers}{ cartographer "cartographer" format.names duplicate$ empty$ 'skip$    {      "," *      " " *      get.bbl.cartographer      *    }  if$}FUNCTION {format.inventors}{ inventor "inventor" format.names duplicate$ empty$ 'skip$    {      "," *      " " *      get.bbl.inventor      *    }  if$}FUNCTION {format.note}{ note empty$    { "" }    { note #1 #1 substring$      duplicate$ "{" =        'skip$        { output.state mid.sentence =          { "l" }          { "u" }        if$        change.case$        }      if$      note #2 global.max$ substring$ * "note" bibinfo.check    }  if$}FUNCTION {format.title}{ title%%duplicate$ empty$ 'skip$%%  { "t" change.case$ }%%if$  "title" bibinfo.check}FUNCTION {format.type}{ type empty$    'skip$    { inbrackets type }    %%{ add.blank "[" type * "]" * }  if$}FUNCTION {output.bibitem}{ outside.brackets 'bracket.state := % urlbst   newline$  "\bibitem{" write$  cite$ write$  "}" write$  newline$  ""  before.all 'output.state :=}FUNCTION {n.dashify}{  't :=  ""    { t empty$ not }    { t #1 #1 substring$ "-" =        { t #1 #2 substring$ "--" = not            { "--" *              t #2 global.max$ substring$ 't :=            }            {   { t #1 #1 substring$ "-" = }                { "-" *                  t #2 global.max$ substring$ 't :=                }              while$            }          if$        }        { t #1 #1 substring$ *          t #2 global.max$ substring$ 't :=        }      if$    }  while$}FUNCTION {word.in}{ bbl.in capitalize  ":" *  " " * }FUNCTION {format.journal.date}{  month "month" bibinfo.check  duplicate$ empty$  year  "year"  bibinfo.check duplicate$ empty$    {      swap$ 'skip$      { "there's a month but no year in " cite$ * warning$ }      if$      *    }    { swap$ 'skip$        {          " " * swap$        }      if$      *      remove.dots    }  if$  duplicate$ empty$    'skip$    {      before.all 'output.state :=    after.sentence 'output.state :=    }  if$}FUNCTION {format.date}{  no.blank.or.punct  ";"   duplicate$ empty$  year  "year"  bibinfo.check duplicate$ empty$    { swap$ 'skip$        { "there's a month but no year in " cite$ * warning$ }      if$      *    }    { swap$ 'skip$        {          swap$          " " * swap$        }      if$      *    }  if$}FUNCTION {format.btitle}{ title "title" bibinfo.check  duplicate$ empty$ 'skip$    {    }  if$}FUNCTION {either.or.check}{ empty$    'pop$    { "can't use both " swap$ * " fields in " * cite$ * warning$ }  if$}FUNCTION {format.bvolume}{ volume empty$    { "" }    { bbl.volume volume tie.or.space.prefix      "volume" bibinfo.check * *      series "series" bibinfo.check      duplicate$ empty$ 'pop$        { swap$ bbl.of space.word * swap$          emphasize * }      if$      "volume and number" number either.or.check    }  if$}FUNCTION {format.number.series}{ volume empty$    { number empty$        { series field.or.null }        { series empty$            { number "number" bibinfo.check }        { output.state mid.sentence =            { bbl.number }            { bbl.number capitalize }          if$          number tie.or.space.prefix "number" bibinfo.check * *          bbl.in space.word *          series "series" bibinfo.check *        }      if$    }      if$    }    { "" }  if$}FUNCTION {is.num}{ chr.to.int$  duplicate$ "0" chr.to.int$ < not  swap$ "9" chr.to.int$ > not and}FUNCTION {extract.num}{ duplicate$ 't :=  "" 's :=  { t empty$ not }  { t #1 #1 substring$    t #2 global.max$ substring$ 't :=    duplicate$ is.num      { s swap$ * 's := }      { pop$ "" 't := }    if$  }  while$  s empty$    'skip$    { pop$ s }  if$}FUNCTION {convert.edition}{ extract.num "l" change.case$ 's :=  s "first" = s "1" = or    { bbl.first 't := }    { s "second" = s "2" = or        { bbl.second 't := }        { s "third" = s "3" = or            { bbl.third 't := }            { s "fourth" = s "4" = or                { bbl.fourth 't := }                { s "fifth" = s "5" = or                    { bbl.fifth 't := }                    { s #1 #1 substring$ is.num                        { s eng.ord 't := }                        { edition 't := }                      if$                    }                  if$                }              if$            }          if$        }      if$    }  if$  t}FUNCTION {format.edition}{ edition duplicate$ empty$ 'skip$    {      convert.edition      output.state mid.sentence =        { "l" }        { "t" }      if$ change.case$      "edition" bibinfo.check      " " * bbl.edition *    }  if$}INTEGERS { multiresult }FUNCTION {multi.page.check}{ 't :=  #0 'multiresult :=    { multiresult not      t empty$ not      and    }    { t #1 #1 substring$      duplicate$ "-" =      swap$ duplicate$ "," =      swap$ "+" =      or or        { #1 'multiresult := }        { t #2 global.max$ substring$ 't := }      if$    }  while$  multiresult}FUNCTION {format.pages}{ pages duplicate$ empty$ 'skip$    { duplicate$ multi.page.check        {          bbl.pages swap$          n.dashify        }        {          bbl.page swap$        }      if$      tie.or.space.prefix      "pages" bibinfo.check      * *    }  if$}FUNCTION {format.journal.pages}{ pages duplicate$ empty$ 'pop$    { swap$ duplicate$ empty$        { pop$ pop$ format.pages }        {          ":" *          swap$          n.dashify          "pages" bibinfo.check          *        }      if$    }  if$}FUNCTION {format.vol.num}{ volume field.or.null  duplicate$ empty$ 'skip$    {      "volume" bibinfo.check    }  if$  number "number" bibinfo.check duplicate$ empty$ 'skip$    {      swap$ duplicate$ empty$        { "there's a number but no volume in " cite$ * warning$ }        'skip$      if$      swap$      "(" swap$ * ")" *    }  if$ *}FUNCTION {format.vol.num.pages}{ volume field.or.null  duplicate$ empty$ 'skip$    {      "volume" bibinfo.check    }  if$  number "number" bibinfo.check duplicate$ empty$ 'skip$    {      swap$ duplicate$ empty$        { "there's a number but no volume in " cite$ * warning$ }        'skip$      if$      swap$      "(" swap$ * ")" *    }  if$ *  format.journal.pages}FUNCTION {format.chapter.pages}{ chapter empty$    'format.pages    { type empty$        { bbl.chapter }		{ type "l" change.case$		  "type" bibinfo.check		}	      if$	      chapter tie.or.space.prefix	      "chapter" bibinfo.check	      * *	      pages empty$		'skip$		{ ", " * format.pages * }	      if$	    }	  if$	}	FUNCTION {format.booktitle}	{	  booktitle "booktitle" bibinfo.check	}	FUNCTION {format.in.ed.booktitle}	{ format.booktitle duplicate$ empty$ 'skip$	    {	      editor "editor" format.names.ed duplicate$ empty$ 'pop$		{		  "," *		  " " *		  get.bbl.editor		  ". " *		  * swap$		  * }	      if$	      word.in swap$ *	    }	  if$	}	FUNCTION {format.in.ed.title}	{ format.title duplicate$ empty$ 'skip$	    {	      editor "editor" format.names.ed duplicate$ empty$ 'pop$		{		  "," *		  " " *		  get.bbl.editor		  ". " *		  * swap$		  * }	      if$	      word.in swap$ *	    }	  if$	}	FUNCTION {empty.misc.check}	{ author empty$ title empty$ howpublished empty$	  month empty$ year empty$ note empty$	  and and and and and	    { "all relevant fields are empty in " cite$ * warning$ }	    'skip$	  if$	}	FUNCTION {format.thesis.type}	{ type duplicate$ empty$	    'pop$	    { swap$ pop$	      "t" change.case$ "type" bibinfo.check    }  if$}FUNCTION {format.tr.number}{    number "number" bibinfo.check  %%type duplicate$ empty$    %%{ pop$ bbl.techrep }    %%'skip$  %%if$  %%"type" bibinfo.check  %%swap$ duplicate$ empty$    %%{ pop$ "t" change.case$ }    %%{ tie.or.space.prefix * * }  %%if$}FUNCTION {format.org.or.pub}{ 't :=  ""  address empty$ t empty$ and    'skip$    {      address "address" bibinfo.check *      t empty$        'skip$        { address empty$            'skip$            { ": " * }          if$          t *        }      if$    }  if$}FUNCTION {format.publisher.address}{ publisher "publisher" bibinfo.warn format.org.or.pub}FUNCTION {format.organization.address}{ organization "organization" bibinfo.check format.org.or.pub}FUNCTION {format.institution.address}{ institution "institution" bibinfo.check format.org.or.pub}% urlbst...% Functions for making hypertext links.% In all cases, the stack has (link-text href-url)%% make 'null' specialsFUNCTION {make.href.null}{  pop$}% make hypertex specialsFUNCTION {make.href.hypertex}{   "\special {html:<a href=" quote$ *  swap$ * quote$ * "> }" * swap$ *  "\special {html:</a>}" *}% make hyperref specialsFUNCTION {make.href.hyperref}{   "\href {" swap$ * "} {" * swap$ * "}" *}FUNCTION {make.href}{ hrefform #2 =    'make.href.hyperref      % hrefform = 2    { hrefform #1 =        'make.href.hypertex  % hrefform = 1        'make.href.null      % hrefform = 0 (or anything else)      if$    }  if$}FUNCTION {format.url}{ url empty$    { "" }      { hrefform #1 =          { % special case -- add HyperTeX specials            urlintro "\url{" url * "}" * url make.href.hypertex * }          { urlintro "\url{" * url * "}" * }       if$     }  if$}FUNCTION {format.eprint}{ eprint empty$    { "" }    { eprintprefix eprint * eprinturl eprint * make.href }  if$}FUNCTION {format.doi}{ doi empty$    { "" }    { doiprefix doi * doiurl doi * make.href }  if$}% Output a URL.  We can't use the more normal idiom (something like% `format.url output'), because the `inbrackets' within% format.lastchecked applies to everything between calls to `output',% so that `format.url format.lastchecked * output' ends up with both% the URL and the lastchecked in brackets.FUNCTION {output.url}{ url empty$    'skip$     { new.block       format.url output      format.lastchecked output     }  if$}FUNCTION {output.web.refs}{  new.block  output.url  addeprints eprint empty$ not and    { format.eprint output.nonnull }    'skip$  if$  adddoiresolver doi empty$ not and    { format.doi output.nonnull }    'skip$  if$%  addeprints%    { eprint empty$%        'skip$%        { format.eprint output.nonnull }%      if$%    }%    'skip$%  if$}% Webpage entry type.% Title and url fields required;% author, note, year, month, and lastchecked fields optionalSTRINGS {database}FUNCTION {webpage}{ output.bibitem  author empty$    { editor empty$        'skip$  % author and editor both optional        { format.editors output.nonnull }      if$    }    { editor empty$        { format.authors output.nonnull }        { "can't use both author and editor fields in " cite$ * warning$ }      if$    }  if$%  author empty$%    'skip$%    { format.authors output.nonnull }%  if$  new.block  format.title "title" output.check  journal empty$    {      format.type "type" output.check      publisher empty$        'skip$        { format.publisher.address output }      if$      "database on the Internet" 'database :=      type database =        { format.journal.date "year" output.check }        { format.date "year" output.check }      if$      lastchecked empty$        'skip$        { format.lastchecked output }      if$      new.block      part empty$        'skip$        { part output }      if$      pages empty$        'skip$        { pages bracket.check output }      if$    }    { journal      remove.dots      "journal" bibinfo.check      "journal" output.check      format.type "type" output.check      format.journal.date "year" output.check      lastchecked empty$        'skip$        { format.lastchecked output	  ";" no.blank.or.punct output	}      if$      no.blank.or.punct format.vol.num output      pages empty$        'skip$	{ ":" no.blank.or.punct output	  no.blank.or.punct pages bracket.check output	}      if$      new.block    }  if$  format.url "url" output.check  new.block  note output  fin.entry}% ...urlbst to hereFUNCTION {misc}{ output.bibitem  format.authors "author" output.check  format.editors "author and editor" output.check  format.title "title" output.check  type missing$    { skip$ }    { format.type "type" output.check }    %%{ inbrackets type output }  if$  new.block  format.publisher.address output  format.date "year" output.check  new.block  format.note output  new.block  howpublished new.block.checka  howpublished "howpublished" bibinfo.check output  output.web.refs  % urlbst  fin.entry  empty.misc.check}FUNCTION {article}{ output.bibitem  format.authors "author" output.check  organization empty$    'skip$    { author empty$        {          format.organizations "organization" output.check	}	{	  "; " *	  no.blank.or.punct          format.organizations "organization" output.check	}      if$    }  if$  new.block  format.title "title" output.check  type missing$    { skip$ }    { format.type "type" output.check }  if$  new.block  journal  remove.dots  "journal" bibinfo.check  "journal" output.check  format.journal.date "year" output.check  add.semicolon  format.vol.num.pages output  new.block  format.note output  output.web.refs  % urlbst  fin.entry}FUNCTION {book}{ output.bibitem  author empty$    { editor empty$        { format.organizations "organization" output.check }        { format.editors "author and editor" output.check }      if$    }     { format.authors output.nonnull      "author and editor" editor either.or.check    }  if$  new.block  format.btitle "title" output.check  format.bvolume output  new.block  format.edition output  new.sentence  author empty$ not  editor empty$ not  and    { format.editors "author and editor" output.check }      'skip$  if$  format.number.series output  format.publisher.address output  format.date "year" output.check  new.block  format.note output  output.web.refs  % urlbst  fin.entry}FUNCTION {booklet}{ misc }FUNCTION {dictionary}{ output.bibitem  format.booktitle "booktitle" output.check  format.bvolume output  new.block  format.edition output  new.sentence  format.publisher.address output  format.date "year" output.check  format.btitle "title" output.check  add.semicolon  add.blank  format.pages "pages" output.check  new.block  format.note output  output.web.refs  % urlbst  fin.entry}FUNCTION {inbook}{ output.bibitem  format.authors "author" output.check  new.block  chapter "chapter" output.check  new.block  format.in.ed.title "title" output.check  format.bvolume output  format.edition output  new.sentence  format.number.series output  format.publisher.address output  format.date "year" output.check  date.block  add.blank  format.pages "pages" output.check  new.block  format.note output  output.web.refs  % urlbst  fin.entry}FUNCTION {incollection}{ output.bibitem  format.authors "author" output.check  new.block  format.title "title" output.check  new.block  format.in.ed.booktitle "booktitle" output.check  format.bvolume output  format.edition output  new.sentence  format.number.series output  format.publisher.address output  format.date "year" output.check  date.block  add.blank  format.pages "pages" output.check  new.block  format.note output  output.web.refs  % urlbst  fin.entry}FUNCTION {inproceedings}{ output.bibitem  format.authors "author" output.check  new.block  format.title "title" output.check  new.block  format.in.ed.booktitle "booktitle" output.check  format.bvolume output  new.sentence  format.number.series output  publisher empty$    { format.organization.address output }    { organization "organization" bibinfo.check output      format.publisher.address output    }  if$  format.date "year" output.check  date.block  add.blank  format.pages "pages" output.check  new.block  format.note output  output.web.refs  % urlbst  fin.entry}FUNCTION {conference}{inproceedings}FUNCTION {manual}{misc}FUNCTION {phdthesis}{ output.bibitem  format.authors "author" output.check  new.block  format.btitle  "title" output.check  format.type "type" output.check  new.block  school "school" bibinfo.warn output  address "address" bibinfo.check output  format.date "year" output.check  new.block  format.note output  output.web.refs  % urlbst  fin.entry}FUNCTION {mastersthesis}{phdthesis}FUNCTION {proceedings}{ output.bibitem  editor empty$    { organization "organization" bibinfo.check output    }    { format.editors output.nonnull }  if$  new.block  format.btitle "title" output.check  format.bvolume output  editor empty$    { publisher empty$        'skip$        {          new.sentence          format.number.series output          format.publisher.address output        }      if$    }    { publisher empty$        {          new.sentence          format.organization.address output }        {          new.sentence          organization "organization" bibinfo.check output          format.publisher.address output        }      if$     }  if$      format.date "year" output.check  new.block  format.note output  output.web.refs  % urlbst  fin.entry}FUNCTION {techreport}{ output.bibitem  format.authors "author" output.check  new.block  format.title  "title" output.check  new.block  format.institution.address output  format.date "year" output.check  format.tr.number output.nonnull  new.block  format.note output  output.web.refs  % urlbst  fin.entry}FUNCTION {map}{ output.bibitem  format.cartographers "cartographer" output.check  new.block  format.title  "title" output.check  format.type "type" output.check  new.block  format.publisher.address output  format.date "year" output.check  new.block  format.note output  output.web.refs  % urlbst  fin.entry}FUNCTION {patent}{ output.bibitem  format.inventors "inventor" output.check  "; " *  no.blank.or.punct  format.assignees "assignee" output.check  new.block  format.title  "title" output.check  new.block  format.tr.number output.nonnull  format.date "year" output.check  new.block  format.note output  output.web.refs  % urlbst  fin.entry}FUNCTION {unpublished}{ output.bibitem  format.authors "author" output.check  new.block  format.title "title" output.check  format.date output  new.block  format.note "note" output.check  output.web.refs  % urlbst  fin.entry}FUNCTION {default.type} { misc }READSTRINGS { longest.label }INTEGERS { number.label longest.label.width }FUNCTION {initialize.longest.label}{ "" 'longest.label :=  #1 'number.label :=  #0 'longest.label.width :=}FUNCTION {longest.label.pass}{ number.label int.to.str$ 'label :=  number.label #1 + 'number.label :=  label width$ longest.label.width >    { label 'longest.label :=      label width$ 'longest.label.width :=    }    'skip$  if$}EXECUTE {initialize.longest.label}ITERATE {longest.label.pass}FUNCTION {begin.bib}{ preamble$ empty$    'skip$    { preamble$ write$ newline$ }  if$  "\begin{thebibliography}{"  longest.label  * "}" *  write$ newline$}EXECUTE {begin.bib}EXECUTE {init.config.constants}EXECUTE {init.state.consts}ITERATE {call.type$}FUNCTION {end.bib}{ newline$  "\end{thebibliography}" write$ newline$}EXECUTE {end.bib}%% End of customized bst file%%%% End of file `vancouver.bst'.
